# Prophylaxis with erythropoietin versus placebo reduces acute kidney injury and neutrophil gelatinase-associated lipocalin in patients undergoing cardiac surgery: a randomized, double-blind controlled trial

**DOI:** 10.1186/1471-2369-14-136

**Published:** 2013-07-05

**Authors:** Adis Tasanarong, Soodkate Duangchana, Sangduen Sumransurp, Boonlawat Homvises, Opas Satdhabudha

**Affiliations:** 1 Nephrology Unit, Department of Medicine, Faculty of Medicine, Thammasat University (Rangsit Campus), Khlong Nung, Khlong Luang, Pathumthani 12121, Thailand; 2 Cardiovascular and Thoracic Unit, Department of Surgery, Faculty of Medicine, Thammasat University (Rangsit Campus), Khlong Nung, Khlong Luang, Pathumthani 12121, Thailand

**Keywords:** Erythropoietin, Acute kidney injury, Neutrophil Gelatinase-Associated Lipocalin (NGAL), Cardiac surgery

## Abstract

**Background:**

Cardiac surgery-associated acute kidney injury (CSA-AKI) is a common complication following coronary bypass graft (CABG) surgery. Multi-factorial causes of CSA-AKI involve oxidative stress and inflammation. Erythropoietin (EPO) has been shown from many studies to have a reno-protective effect. The present study was conducted to examine the role of EPO in preventing CSA-AKI.

**Methods:**

This prospective, randomized, double-blind, placebo-controlled trial was conducted in the Cardiovascular and Thoracic Unit. One hundred patients randomly received either 200 U/kg of rHuEPO (n = 50) or saline (n = 50) intravenously three days before operation, and rHuEPO 100 U/kg or saline at operation time. The serum creatinine (SCr), estimated glomerular filtration rate (eGFR) and urine neutrophil gelatinase-associated lipocaline (NGAL) were measured in order to evaluate renal injury following CABG.

**Results:**

The incidence of CSA-AKI was significantly lower in rHuEPO group (14%) when compared with the placebo group (38%; p < 0.01). The mean intensive care unit (ICU) and hospital stays of the rHuEPO group were significantly shorter than the placebo group (p < 0.01). Postoperative increases in SCr and decreases in eGFR were significantly lower in the rHuEPO group than the placebo group (p < 0.05). The mean urine NGAL in rHuEPO group was significantly lower than the placebo group at 3 hr, 6 hr, 12 hr and 18 hr after CABG (p < 0.05), respectively.

**Conclusions:**

Prophylaxis administration with intravenous rHuEPO before cardiac surgery decreased the incidence of CSA-AKI and urine NGAL with reduced days in ICU and hospital in elective CABG patients.

**Trial registration:**

ClinicalTrials.gov: NCT01066351

## Background

Acute kidney injury (AKI) following cardiac surgery is the second most common cause of AKI in critically ill patients [1]. The incidence of cardiac surgery-associated acute kidney injury (CSA-AKI) varies between 7.7 to 42% and is associated with prolonged admission in the intensive care unit (ICU) and longer hospital stay [2,3]. CSA-AKI requiring renal replacement therapy (RRT) following coronary artery bypass grafting (CABG) surgery occurs in approximately 0.7 to 3.5% and is associated with an increase in mortality rate [4,5]. Minimal increase of serum creatinine (SCr) correlated with increase mortality and affected long-term survival after cardiac surgery [6,7]. Multiple causes of CSA-AKI have been proposed, including cardiovascular compromise, prolonged cardiopulmonary bypass (CPB) exposure, hemolysis, hypothermia, decline of renal perfusion, and reperfusion after operation [8]. These causes could induce ischemic reperfusion injury, generate reactive oxygen species and activate inflammatory pathways [9]. Many studies have tried to demonstrate the advantage of various pharmacologic interventions to prevent CSA-AKI such as dopamine, N-acetylcysteine, statin, and fenoldopam but the results have been conflicting [10,11].

Erythropoietin (EPO) is a 30 kDa glycoprotein produced by kidney that regulates red blood cell production in the bone marrow [12]. Recombinant human erythropoietin (rHuEPO) has been considered as a novel reno-protective therapy beyond the hematopoietic effect in AKI and chronic kidney disease (CKD) [13,14]. rHuEPO plays an important role as an anti-apoptotic, anti-inflammation and anti-oxidant properties in many models of kidney disease [15-17]. In experimental models of AKI, rHuEPO administration before, during or even after injury has attenuated kidney damage in AKI [12,18] and has slowed the progression during chronic kidney injury [18]. However, the few clinical trials in humans have produced the conflicting results regarding to the reno-protective effect of rHuEPO in AKI. Similarly, previous clinical trials demonstrated that the early anemia treatment in CKD patients with rHuEPO did not produce a consistent effect on slowing the progression of CKD [19-22].

Because of the different diagnostic criteria of CSA-AKI that was based on SCr and/or urine output [23,24], the use of these criteria still have many limitations, especially in the reliability of SCr in AKI patients and lack of real-time estimation of glomerular filtration rate (eGFR). Furthermore, SCr is an unreliable biomarker during AKI in kidney function because many factors can affect SCr level including creatinine generation by muscle catabolism and diet, age, hydration status and renal tubular secretion [25]. Changes in SCr are delayed in time after kidney injury [26]. Thus, the development of novel AKI related biomarkers could help the physicians in the early detection of CSA-AKI [26,27].

In the last decade, many publications have evaluated the accuracy and reliability of novel urinary and serum biomarkers like neutrophil gelatinase-associated lipocalin (NGAL) for the early detection and/or predict the prognosis of AKI [28]. However, there remains the space between the damage in biological processes and the clinical presentation during AKI, so such markers have not yet found a place in routine clinical practice [29,30]. Although, none novel biomarkers has the consensus to approach in clinical decision making in diagnosis patients with AKI, but NGAL detected patients with subclinical AKI in spite of unchanged SCr [31]. In addition, delayed diagnosis of AKI based on SCr changing could explain some negative results of the interventions in many clinical trials [32]. NGAL is a 25 kDa protein covalently bound to gelatinase in neutrophils and is usually expresses at very low levels in several human tissues, including kidney, lung, stomach, and colon [33,34]. During AKI, NGAL expression is markedly increased in the injured distal nephron epithelia, and is not reabsorbed by the damaged proximal tubules resulting in an elevation of urinary NGAL [35]. NGAL protein was easily detected in the blood and urine soon after AKI in animal and human diseases [36,37] and used in the detection of CSA-AKI in patients undergoing cardiac surgery [38,39].

Given the uncertainty of the use of rHuEPO for renal protection and the promising use of NGAL for detecting AKI, we conducted a prospective, randomized, double-blind, placebo-controlled trial to evaluate the reno-protective effect of rHuEPO when started three days prior to the onset of cardiac surgery and at the operation time. This early start is intended as a means of preventing AKI in elective CABG patients. The advantage of rHuEPO was evaluated on the incidence of CSA-AKI, clinical outcomes and changing of urine NGAL.

## Methods

### Patient population

Study patients were aged at least 18 years who were scheduled for elective CABG using the CPB technique at Thammasat Chalerm Prakiat Hospital during the period from January 2010 to March 2011 were included in the study. The protocol was approved by the Ethics Committee of the Faculty of Medicine at Thammasat University. All patients provided written, informed consent to participate in the study. Patients with AKI before randomization, CKD stage 5 or unstable renal function (as evidenced by a change in SCr of ≥ 0.3 mg/dL, or ≥ 50%, within 14 days prior to the study), using the nephrotoxic drugs and/or contrast media administration within two weeks before operation and using rHuEPO prior to CABG were excluded. Subjects were also excluded if they had a known allergy to any of the rHuEPO, suffered from congestive heart failure, cardiogenic shock or emergent CABG. The study was done in full compliance with the Declaration of Helsinki. This trial was registered in the Protocol Registration System (ClinicalTrials.gov Identifier: NCT01066351).

### Study protocol

This was a single center with balanced randomization 1:1 ratios, double-blind, placebo controlled trial. Treatment assignment among the two groups was determined by blocked randomization. After recruitment, three days before the operation, sealed envelopes containing the allocation group were opened by nurses who did not participate in the study. All patients were enrolled into this study were randomized into two groups: the patients who received rHuEPO (rHuEPO group) and 0.9% saline (placebo group). The same nurse and perfusionist prepared the treatments that were blindly given to the research coordinator. Patients and investigators were also blinded to group assignment. Pairs of identical 0.3 ml syringes containing either rHuEPO (5,000 U, Recormon, Roche) or saline were prepared and stored. Patients received an intravenous dose of 200 U/kg or saline three days before operation and either 100 U/kg of rHuEPO or saline intravenously at the operation time.

Demographic and baseline characteristics including patient age, sex, blood pressure, and co-morbidities were collected at time of group assignment. In addition, intra-operative data, operation time, arterial clamp time, the amount of fluid or blood infused and the urine output were collected.

### Urine and serum sample collections and storage

Blood samples were obtained for the measurement of complete blood count (CBC) and percent of reticulocyte count three days before the operation (baseline), 3 to 6 hr before the operation, and daily CBC for five days post-operation. Baseline SCr was measured at 6 to 12 hr prior to operation and post-operative daily at least for five days in all patients. eGFR was calculated using the Cockroft-Gault equation. All laboratory parameters were performed in a single, hospital-based laboratory using standard methods. Serial urine samples were collected at baseline and 3, 6, 12, 18 and 24 h after operation. Samples were centrifuged at 2,000 g for 5 min and the supernatants stored at −70°C until assayed.

Urinary NGAL concentration was measured using a commercial ELISA kit (Antibody Shop, Gentofte, Denmark), following the manufacturer’s instructions. All urine specimens were diluted to achieve concentration for optimal density before performing the ELISA assay to fit the concentrations of respective NGAL protein in the linear range of the standard curve. The inter-assay and intra-assay coefficients of variation for NGAL were < 5%. The measurements were made in duplicate and in a blinded fashion.

### Study end points

The primary endpoint of this study was the incidence of CSA-AKI in rHuEPO compared with placebo group. The definition of CSA-AKI is defined as a ≥ 0.3 mg/dl or ≥ 50% increase in SCr levels from baseline within the first 48 hr post-operation according to the newer criteria of AKI from KDIGO guideline 2012. Secondary endpoints consisted of comparative changes in SCr, eGFR and urine NGAL during the first three postoperative days, postoperative complications, length of stay in the intensive care unit (ICU) and hospital, a requirement for renal replacement therapy (RRT) and all causes hospital mortality between rHuEPO and placebo groups.

### Sample size

The sample size was calculated to demonstrate a reduction in the incidence of CSA-AKI from 40% in the placebo group to 15% in the rHuEPO group. With the use of a two-sided X^2^ test with a significance level of 0.05 and a power of 90%, the sample size in each group was 65 patients. However, the statistic significant between both groups become since the population size in each group was 50 patients. Therefore, a total of 100 patients were randomized in the present study.

### Statistical analysis

Data were expressed as a mean ± SD for continuous variables and as percentages for discrete variables. Continuous data were analyzed by the Student’s t test for equal variance or Mann–Whitney test for unequal variance, and categorical valuables were investigated by the Pearson χ^2^ or Fisher’s exact test. A two-sided p value < 0.05 was considered significant. Two-way analysis of variance was utilized to compare continuous variables over time between the two groups with Bonferroni post-hoc test for each time point. Statistical analyses were performed using SPSS (Version 15.0. for Windows; SPSS, Inc.) and significance was assigned when *p* values < 0.05.

## Results

**Figure 1:**
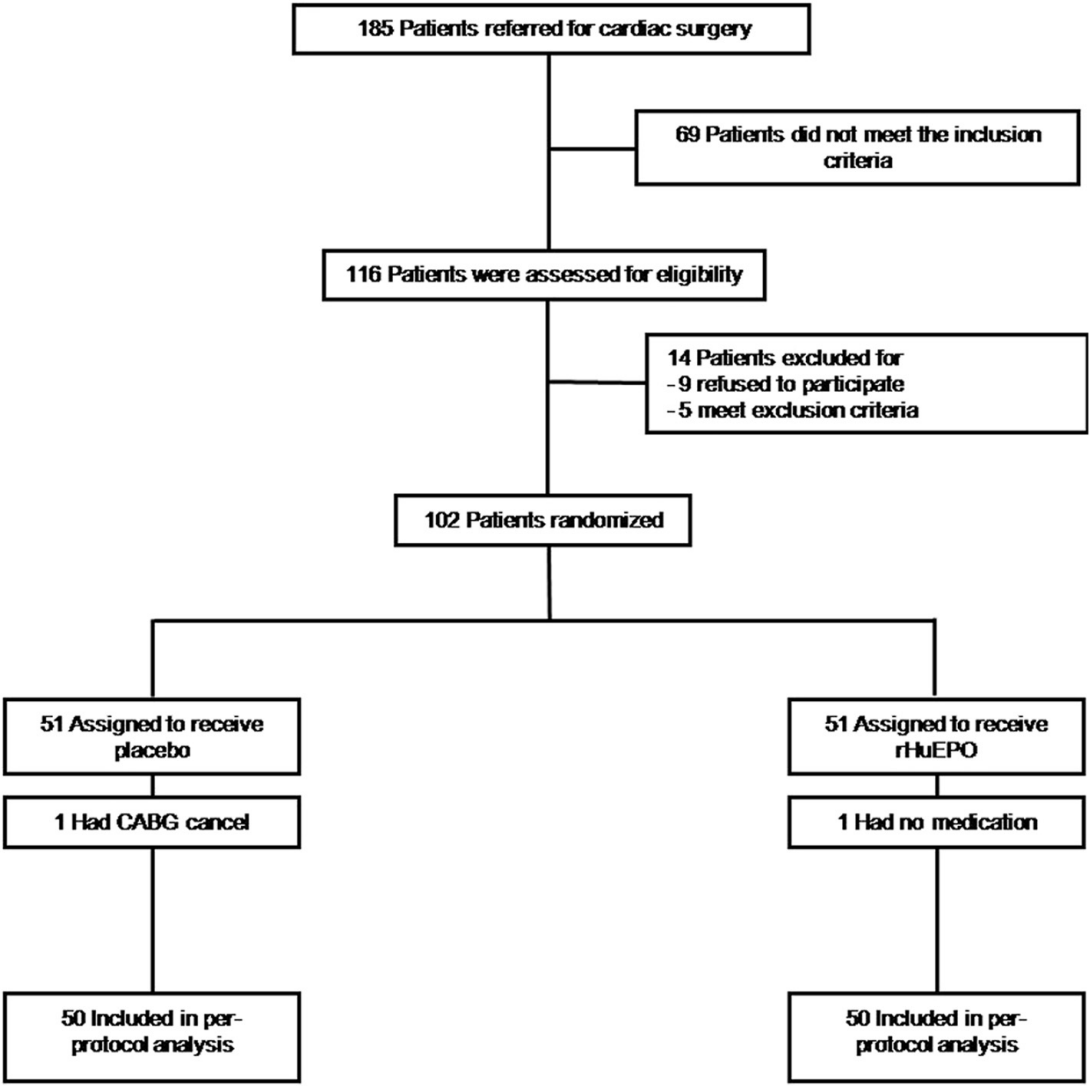
Flow diagram on enrollment of patients into the study.

A total of 185 patients referred for cardiac surgery were screened between January 2010 and March 2011 (Figure 1). Of these patients, 116 patients met the inclusion criteria and were enrolled in the study. Fourteen patients were excluded from the study because nine patients refused to participate, two patients suffered from congestive heart failure, and three patients developed AKI. Finally, 102 patients were randomized and allocated to one of the two treatment arms between the placebo and rHuEPO group. One patient in the rHuEPO and one from the placebo group were excluded because of the lack of medical treatment and a CABG cancelation. Consequently, 100 patients received their assigned treatments to the placebo (n = 50) or rHuEPO (n = 50) group. The baseline characteristics and intra-operative information for these study participants are shown in Table 1. There were no statistically significant differences between the two groups regarding clinical characteristics, especially existing co-morbidities and preoperative medications. In addition, preoperative hemoglobin, hematocrit, reticulocyte count, SCr and eGFR were comparable between the two groups. The operation time, arterial clamp time, central venous pressure, fluid intake and urine output during operation were equivalent between both groups.

**Table 1:** Baseline characteristics and intra-operative data of enrolled patients randomized to placebo or rHuEPO group

	**Placebo group (n = 50)**	**rHuEPO group (n = 50)**	**p value**
Age (years)	60 ± 16	63 ± 16	0.39
Sex: Male (%)	52	62	0.31
Underlining Disease:			
Diabetes mellitus (%)	34	27	0.54
Hypertension (%)	63	79	0.15
Dyslipidemia (%)	38	36	0.92
**Preoperative medication:**			
ACE inhibitor (%)	44	46	0.72
Angiotensin II recertor blocker (%)	22	24	0.86
Beta blocker (%)	46	50	0.62
Calcium channel blocker (%)	34	36	0.68
Diuretic (%)	26	30	0.24
Platelet inhibitor (%)	74	82	0.16
Statin (%)	62	60	0.84
Serum creatinine (SCr), mg/dL	1.05 ± 0.45	1.05 ± 0.27	0.94
eGFR (mL/min/1.73 m^2^)	67 ± 33	64 ± 29	0.44
eGFR > 60 (%)	48	38	0.48
eGFR < 60 (%)	52	62	0.32
Hemoglobin (g/dL)	12.2 ± 1.9	12.3 ± 1.7	0.72
Hematocrit (%)	36.2 ± 6.6	37.1 ± 5.2	0.45
Reticulocyte count (%)	1.19 ± 0.66	1.11 ± 0.63	0.53
**Intra-operative data:**			
Operation time (minutes)	353 ± 76	341 ± 88	0.44
Arterial clamp time (minutes)	87 ± 31	82 ± 38	0.52
Central venous pressure (cm H_2_O)	4.81 ± 4.07	4.52 ± 3.93	0.72
Intraoperative intake (mL)	1,628 ± 660	1,530 ± 477	0.55
Intraoperative output (mL)	958 ± 673	1172 ± 973	0.34
Intraoperative urine output (mL/kg/hour)	2.89 ± 1.73	2.96 ± 2.46	0.89

eGFR indicates estimated glomerular filtration rate.

**Figure 2:**
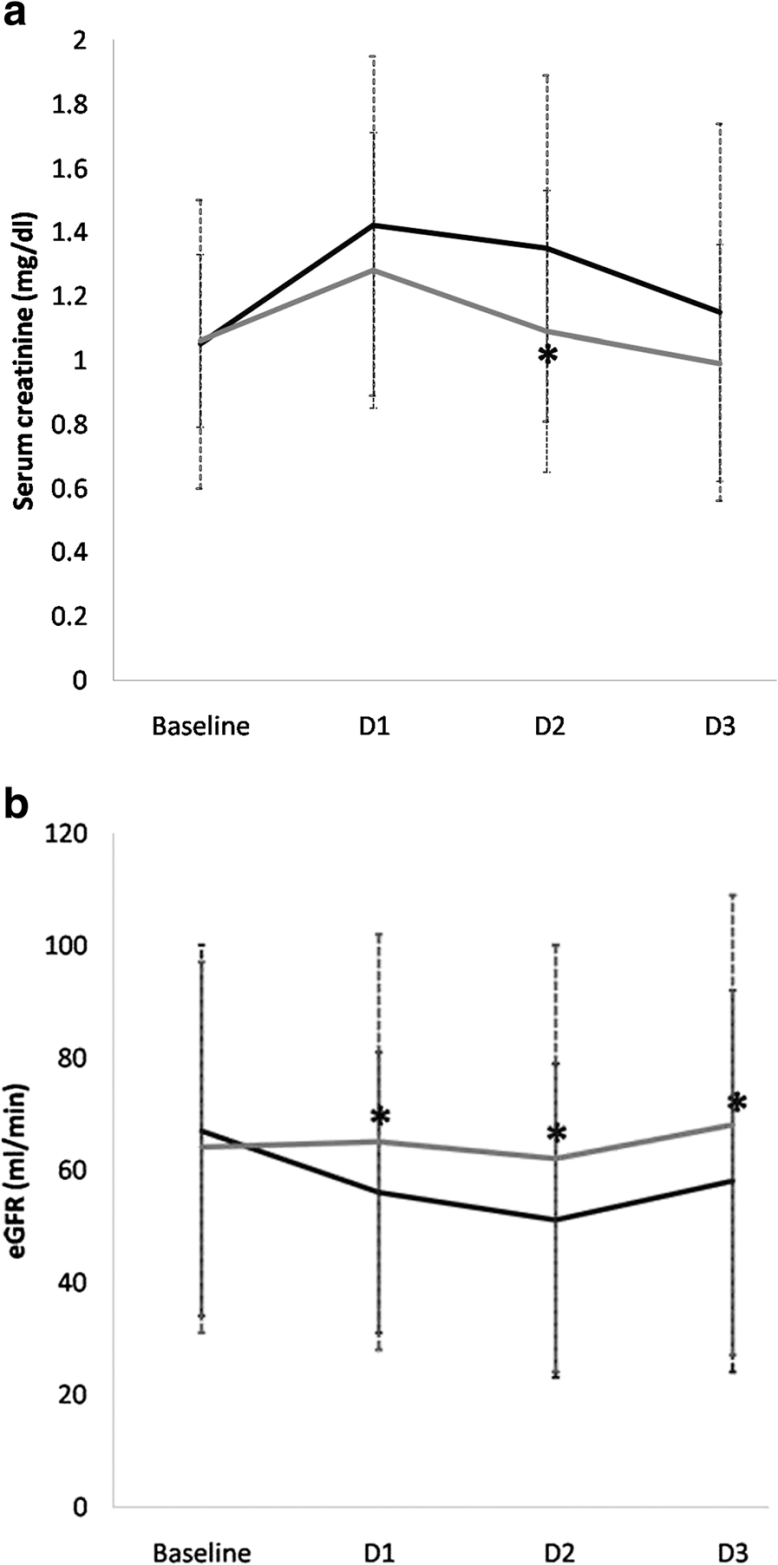
**Mean changes of SCr (2A) and eGFR (2B) at day 1 (D1), day 2 (D2), and day 3 (D3) after operation compared with baseline in patients randomized to placebo (black line) or rHuEPO (gray line).** *p < 0.05 compared between the placebo and rHuEPO group.

The change in reticulocyte count, hematocrit, SCr and eGFR are shown in Table 2. Baseline reticulocyte count was similar between the two groups. There was a significant increase in the percent reticulocyte count following administration of the first dose of rHuEPO in rHuEPO group (1.11 ± 0.63 to 1.6 ± 0.82; p < 0.01) while no significant change occurred in the placebo group (1.19 ± 0.66 to 1.12 ± 0.56) at operative day. There was no significant difference between the two groups in baseline and postoperative hematocrit. A comparison of the two groups, baseline SCr and eGFR showed no significant differences. In the placebo group, SCr was higher than the baseline at 24, 48 and 72 hr after operation. In contrast, SCr in the rHuEPO group was higher than the baseline at 24 hr but turned down like the baseline at 48 hr and was lower than the baseline at 72 hr after operation (Figure 2A). Moreover, SCr at 48 hr post-operation in the placebo group was significantly higher than the rHuEPO group (p < 0.05), (Table 2). In the placebo group, eGFR was lower than the baseline at 24, 48 and 72 hr after operation (p < 0.05) but eGFR in rHuEPO group was no significant change from the baseline at 24, 48, and 72 hr after operation (Figure 2B). In addition, eGFR was significantly lower in the placebo than the rHuEPO group at 24, 48 and 72 hr after operation (p < 0.05), respectively (Table 2).

**Table 2:** Baseline reticulocyte count, hematocrit, serum creatinine and eGFR with changes after cardiac surgery in patients randomized to placebo or rHuEPO group

	**Placebo group (n = 50)**	**rHuEPO group (n = 50)**	**p value**
Reticulocyte count, %			
Baseline	1.19 ± 0.66	1.11 ± 0.63	0.53
Operative day (3 days after first dose)	1.12 ± 0.56	1.60 ± 0.82	<0.01
Hematocrit (%)			
Baseline	36.7 ± 5.4	37.1 ± 5.2	0.72
Day 0 Pre-operation	36.8 ± 5.8	38.0 ± 5.2	0.31
Day 0 Post-operation	27.8 ± 4.0	29.4 ± 4.9	0.08
Day 1	32.3 ± 3.1	32.7 ± 3.1	0.45
Day 2	32.3 ± 3.4	32.9 ± 3.5	0.4
Day 3	32.3 ± 3.6	33.5 ± 3.9	0.17
Serum creatinine (SCr), mg/dL	1.05 ± 0.45	1.06 ± 0.25	0.83
Baseline			
Day 1 after CABG	1.42 ± 0.53	1.28 ± 0.46	0.25
Day 2 after CABG	1.35 ± 0.44	1.06 ± 0.42	<0.05
Day 3 after CABG	1.18 ± 0.62	0.96 ± 0.38	0.15
eGFR, mL/min/1.73 m^2^			
Baseline	67 ± 33	64 ± 29	0.44
Day 1 after CABG	56 ± 25	65 ± 27	<0.05
Day 2 after CABG	51 ± 28	62 ± 25	<0.05
Day 3 after CABG	58 ± 24	68 ± 26	<0.05

eGFR indicates estimated glomerular filtration rate.

Primary and secondary endpoints are shown in Table 3. CSA-AKI occurred in 26% in the present study. CSA-AKI developed 38% in the placebo group compared with 14% in the rHuEPO group (p < 0.01). Postoperative complications were similar between the two groups. The mean ICU and hospital stay of the rHuEPO group were 4 ± 1 and 11 ± 2 days, which were significantly shorter than the placebo group 7 ± 4 and 17 ± 9 days (p < 0.01), respectively. Two patients in the placebo group required RRT but none in the rHuEPO group during hospital stay. Two patients in the placebo group died in the hospital from sepsis, but no deaths occurred in the rHuEPO group. There was no hypertension, symptomatic thrombosis, myocardial infarction, stroke, seizures or other serious adverse events in the patients who received the rHuEPO. While, there were no significant differences between the rHuEPO and placebo groups regarding incidence of adverse events.

**Table 3:** The study outcomes in patients randomized to placebo or rHuEPO group

	**Placebo group (n = 50)**	**rHuEPO group (n = 50)**	**p value**
**Incidence of AKI n (%)**	19 (38)	7 (14)	<0.01
eGFR > 60 mL/min/1.73 m^2^	6/24 (25)	1/19 (5.26)	0.11
eGFR < 60 mL/min/1.73 m^2^	13/26 (50)	6/31 (19.35)	<0.05
**Postoperative complication:**			
Bleeding n (%)	2 (4)	1 (2)	0.56
Infection n (%)	4 (8)	2 (4)	0.41
Stroke n (%)	1 (2)	0 (0)	0.32
Cardiac arrhythmia n (%)	7 (14)	6 (12)	0.77
Myocardial infarction n (%)	1 (2)	0 (0)	0.32
Hemodialysis n (%)	2 (4)	0 (0)	0.16
**Hospital stay and mortality**			
ICU stay (days)	7 ± 4	4 ± 1	<0.01
Hospital stay (days)	17 ± 9	11 ± 2	<0.01
Hospital deaths n (%)	2 (4)	0 (0)	0.16

eGFR indicates estimated glomerular filtration rate.

ICU indicates intensive care unit.

Baseline and post-operative urine NGAL levels were shown in Table 4. Baseline urine NGAL concentrations were similar in patients between both groups but became higher than baseline at all time points within the first 24 hours in both groups. The mean urine NGAL concentrations in the rHuEPO group were significantly lower than the placebo group at 3 hr (p < 0.05), 6 hr (p < 0.01), 12 hr (p < 0.01) and 18 hr (p < 0.05) after operation. In patients who develop CSA-AKI, the urine NGAL in rHuEPO group were also significantly lower than the placebo group at all postoperative time points (p < 0.05). While, there was no difference in urine NGAL in patients who did not develop CSA-AKI between both groups.

**Table 4:** Mean urine NGAL concentrations before surgery (baseline), 3 hr, 6 hr, 12 hr, 18 hr, and 24 hr after operation for all patients and stratified by the presence of acute kidney injury

**Urine NGAL (ng/ml)**	**Baseline**	**3 hr**	**6 hr**	**12 hr**	**18 hr**	**24 hr**
**All patients**						
Placebo group (n = 50)	61 ± 42	164 ± 144	174 ± 134	164 ± 113	137 ± 95	122 ± 87
rHuEPO group (n = 50)	55 ± 51	105 ± 76*	109 ± 64^#^	114 ± 67^#^	101 ± 62*	93 ± 56
**Patients with acute kidney injury**						
Placebo group (n = 19)	65 ± 23	281 ± 151	296 ± 140	271 ± 81	207 ± 107	173 ± 105
rHuEPO group (n = 7)	62 ± 28	176 ± 41*	167 ± 40^#^	164 ± 57^#^	128 ± 55*	101 ± 52*
**Patients without acute kidney injury**						
Placebo group (n = 31)	58 ± 50	99 ± 89	106 ± 66	104 ± 79	98 ± 60	94 ± 61
rHuEPO group (n = 43)	54 ± 54	93 ± 75	100 ± 64	106 ± 66	97 ± 64	92 ± 58

Data are expressed as mean ± S.D.

Significant differences by Bonferroni post-hoc test of ANOVA.

*p < 0.05, ^#^p < 0.01 compared between placebo and rHuEPO group.

## Discussion

The present study is the first clinical trial that has assessed the prophylactic regimen of intravenous administration of rHuEPO compared with placebo at three days before and immediate operation time in the preventing of CSA-AKI. This prospective, double-blind, randomized, and placebo-controlled trial has shown that prophylaxis with rHuEPO reduced the incidence of CSA-AKI, decreased the length of ICU and hospital stays and attenuated renal injury as assessed by the sensitive biomarker urine NGAL. Moreover, the increase in SCr and the decline in eGFR post-operation were less in the patients with rHuEPO prophylaxis.

Although, many therapeutic prevention strategies have been investigated in clinical trial [10,11], but none protocol has been proven the effective to preventing CSA-AKI. Beyond the anti-anemic effect, the benefit of EPO in protecting the kidneys was demonstrated to be anti-apoptosis, anti-inflammation and anti-oxidant [15-17]. EPO treatment has reno-protective properties in the experimental model of renal ischemic reperfusion injury when given before, during or even after the injury [40]. In the present study, the advantage of rHuEPO prophylaxis was demonstrated by improve the clinical outcomes and diminish urine NGAL within the first three hours following operation, especially in patients who developed CSA-AKI. Patients with rHuEPO prophylaxis experienced fewer post-operative complications, no needed RRT and no deaths, although numbers were too small to show statistically significant differences with the placebo group. A larger clinical trial is needed to assess if rHuEPO confers a survival advantage.

Our outcomes are in agreement with the recent study by Song et al. [41] who shown that the incidence of CSA-AKI in patients treated with high dose of rHuEPO at the time of anesthetic induction was significantly lower when compared with the saline infusion in the patients undergoing elective CABG. However, administration with rHuEPO in the Korean study did not decreased the duration of ICU and hospital stays, and there were no differences in rates of RRT and death post-cardiac surgery. A part of protocol that similar between the present and the Korean study was time to inject rHuEPO immediately following induction of anesthesia before cardiac surgery. A recent study demonstrated that acute systemic and local inflammatory response after cardiac surgery is associated with periopertive AKI [42]. The anti-inflammatory effects of rHuEPO explain its reno-protective effect and preoperative rHuEPO has also been shown to attenuate myocardial ischemic reperfusion injury by inhibiting the systemic inflammatory response [43]. Therefore, this might be the time to get ready for the anti-inflammatory effect of rHuEPO before ischemic reperfusion injury during operation that induces local and systemic inflammatory response. The main difference between our study and the Korean study was the additional administration of rHuEPO three days before cardiac surgery which may explain the excellent results in term of prevent CSA-AKI and clinical outcomes. One could hypothesize that improve anti-oxidant property by rHuEPO administration since three days before ischemic reperfusion injury. The anti-oxidant effect of EPO has been proposed in many mechanisms [17]. The important mechanism is EPO increases the number of circulating young red blood cells (RBC), which raise the level of erythrocyte anti-oxidative enzymes [44]. The increase in circulating young RBC was demonstrated by the improvement of the reticulocyte count which peaks three to four days after rHuEPO injection [45]. Thus, rHuEPO administration three to four days prior to cardiac surgery may be the optimal time to start rHuEPO and a further dose at operation will provide continued anti-inflammatory effect for three to four postoperative days.

Our results contrast with those of two previous studies. Early treatment with high dose rHuEPO compared with placebo following a rise in urine gamma glutamyl transpeptidase and alkaline phosphatase after cardiac surgery by Endre et al. [46] demonstrated no differences in changes in SCr from the baseline at 7 days, the incidence of CSA-AKI, duration of ICU and hospital stays, and rates of RRT and death. Similarly, study by de Seigneux et al. [47] demonstrated that rHuEPO administration shortly after cardiac surgery was inefficient in preventing CSA-AKI and could not reduce the duration of ICU and hospital stays and death. The disadvantage of rHuEPO infusion in cardiac surgery patients may explain from many reasons. First, treatment with rHuEPO after subclinical renal damage or injury could not be the appropriate time to reverse the inflammatory response from surgery. Second, the dose of rHuEPO may be sub therapeutic to recover the kidney function from AKI process. This would limit the applicability of rHuEPO treatment post-intervention to prevent AKI and support the use of prophylactic preoperative rHuEPO regimen.

Studies in cardiac surgery patients reveal that minimal change in SCr or smallest change in SCr that classified by RIFLE criteria had the correlation with increase length of stay in ICU, mortality and postoperative costs [6,7,48,49]. Thus, many studies have assessed novel biomarkers for the early diagnosis AKI before rises in SCr [28,38,39]. However, conflicting results between the changes in these biological damage detectors and clinical AKI have limited their application in clinical practices [29,30]. Recent study demonstrated that subclinical AKI patients detected by a rise in NGAL without a concomitant rise in SCr increased need of RRT, ICU & hospital stay and hospital mortality [31]. Similarly, increase urine NGAL with changes of microscopic examination on the first day in AKI patients improved clinical assessment for predicting the outcome [50]. These reports could point us to confirm the advantage of NGAL to early detection and predict the outcomes in AKI patients. Because of urine NGAL in the present study was significantly lower in the rHuEPO than placebo group at all time points within first 24 hr after operation. Thus, prophylaxis with rHuEPO could reduce the incidence of CSA-AKI by using clinical criteria and novel biomarker assessment. Lower urine NGAL in patients who received rHuEPO provided the evidence of reno-protective effect and correlated with better clinical outcomes.

The present clinical trial has some limitations. First, this study has only been conducted within a single center. Secondly, the results of the present clinical are not representative of incidences of CSA-AKI in patients with normal renal function and require more sample sizes for an adequate scope of study. Thirdly, the author mention to anti-oxidant effect of rHuEPO prophylaxis that indicates an increase of circulating young RBC. So, next study needs to measure the oxidant and anti-oxidant status in these patients. Fourthly, there is a possibility that a multi-dose of rHuEPO before cardiac surgery may be more effective than a single dose in the prevention of CSI-AKI. However, this situation needs more clinical trial to establish.

## Conclusion

Prophylaxis administration with intravenous rHuEPO three days before and at the time of anesthetic induction in patients undergoing cardiac surgery reduced the incidence of clinically defined CSA-AKI, diminish sensitive biomarker urine NGAL and improve the clinical outcomes. A preconditioning regimen based on high dose rHuEPO administration could be more effective in preventing CSA-AKI. More studies are needed to confirm the usefulness of this regimen and larger studies are needed to assess the long term outcomes.

## Abbreviations

EPO: Erythropoeitin; rHuEPO: Recombinant human erythropoietin; AKI: Acute kidney injury; CSA-AKI: Cardiac surgery-associated acute kidney injury; NGAL: Neutrophil gelatinase-associated lipocalin; CKD: Chronic kidney disease; ICU: Intensive care unit; RRT: Renal replacement therapy; CABG: Coronary artery bypass grafting; SCr: Serum creatinine; CPB: Cardiopulmonary bypass; eGFR: Estimation of glomerular filtration rate; RBC: Red blood cells; CBC: Complete blood count.

## Competing interests

All authors declare that they have no competing interest.

## Authors’ contributions

AT designed the protocol, collected the data, analyzed the results and wrote the manuscript. SD and SS collected the data and recruiting the patients. BH and OS collected the data, recruiting the patients and revised the manuscript. All authors read and approved the final manuscript.

## Pre-publication history

The pre-publication history for this paper can be accessed here:

http://www.biomedcentral.com/1471-2369/14/136/prepub
